# Effect of Selenium and Garlic Extract Treatments of Seed-Addressed Lettuce Plants on Biofortification Level, Seed Productivity and Mature Plant Yield and Quality

**DOI:** 10.3390/plants13091190

**Published:** 2024-04-25

**Authors:** Nadezhda Golubkina, Viktor Kharchenko, Anastasia Moldovan, Marina Antoshkina, Olga Ushakova, Agnieszka Sękara, Vasile Stoleru, Otilia Cristina Murariu, Alessio Vincenzo Tallarita, Maura Sannino, Gianluca Caruso

**Affiliations:** 1 Federal Scientific Vegetable Center, 143072 Moscow, Russia; kharchenkoviktor777@gmail.com (V.K.); nastiamoldovan@mail.ru (A.M.); limont_m@mail.ru (M.A.); ems.vniissok@mail.ru (O.U.); 2 Department of Horticulture, Faculty of Biotechnology and Horticulture, University of Agriculture, 31-120 Krakow, Poland; agnieszka.sekara@urk.edu.pl; 3 Department of Horticulture, “Ion Ionescu de la Brad” Iasi University of Life Sciences, 700440 Iasi, Romania; vstoleru@iuls.ro; 4 Department of Food Technology, “Ion Ionescu de la Brad” Iasi University of Life Sciences, 700440 Iasi, Romania; otilia.murariu@iuls.ro; 5 Department of Agricultural Sciences, University of Naples Federico II, 80055 Portici, Italy; alessio.tallarita@unina.it (A.V.T.); maura.sannino@unina.it (M.S.); gcaruso@unina.it (G.C.)

**Keywords:** lettuce, sodium selenate, garlic extract, seed yield and quality, head yield, antioxidants

## Abstract

The enhancement of the plant seed yield and quality represents the basis of the successful productivity of the deriving crop. The effect of single and combined foliar treatments of lettuce plants with sodium selenate and garlic bulb extract on seed yield and quality and on mature plant biochemical characteristics was investigated using four lettuce cultivars (Bouquet, Picnic, Moskovsky parnikovy and Cavalier). The seed production of plants treated with Se increased by 20–41%, compared to the untreated control plants, while the augmentation was as much as 10–23% and 17–27% under garlic extract and the joint application of Se and garlic, respectively. Garlic extract stimulated the accumulation of Se in lettuce seeds, which rose by 1.21–1.29 times compared to the Se-treated plants. The proline levels in lettuce seeds exceeded the corresponding values recorded in the control ones by 1.32–1.64 times in the case of the Se supply, 1.10–1.47 times upon garlic extract application and 1.09–1.31 times under the combined Se/garlic treatment. All the treatments given to lettuce plants increased the leaf weight by 1.10–1.30 times, compared to the untreated control. The seed Se levels positively correlated with the leaf weight (r = 0.621; *p* < 0.005), chlorophyll (r = 0.672, *p* < 0.002) and total antioxidant activity (AOA; r = 0.730, *p* < 0.001) of plants grown from these seeds. Positive correlations were also recorded between the seed proline content and lettuce plant leaf weight, chlorophyll and AOA (r = 0.868, 0.811 and 0.815, respectively, at *p* < 0.001). Lettuce yield was positively correlated with the leaf AOA, chlorophyll and ascorbic acid content (r = 0.942, 0.921 and 0.665, respectively, at *p* < 0.001). The results indicate high prospects of Na_2_SeO_4_ and garlic extract application to seed-addressed lettuce plants, to improve seed productivity and quality, as well as lettuce yield and quality.

## 1. Introduction

Seed yield and quality are considered a fundamental basis of an agricultural crop’s sustainable production. These characteristics depend on plant genetic peculiarities, nutrition, environmental conditions, seed antioxidant status and storage conditions [1,2]. The enhancement of seed productivity, viability and shelf life is being increasingly targeted in connection with the utilization prospects of various growth stimulators and minerals. In this respect, selenium and garlic extracts reportedly provide special benefits [3]. Indeed, various forms of Se (inorganic salts, nanoparticles and organic derivatives) as well as extracts of different garlic tissues (bulbs, peels and stems) show growth stimulation and antioxidant properties, enhancing plant immunity and nutritional value in different species [4,5,6,7,8,9,10,11]. The latter biological effects are partly connected with the biosynthesis of phytohormones, such as auxins under garlic bulb extract application [12], and heteroauxines, gibberellins and ethylene in the case of Se accumulation in plants [13].

The beneficial effects on the seed priming of Se [9,14,15,16,17] and garlic extracts [18,19] consisted of a significant stimulation of seed germination and plant antioxidant activity as well as reduced lipid peroxidation. Moreover, the combined treatment with Se and garlic peel extract in seed priming was effective on yield and antioxidant accumulation in *Vicia faba* [20].

However, the effect of Se or garlic supply on seed productivity has not been deeply studied so far. In this respect, no significant effect of foliar selenate and selenite application was recorded on rice grain productivity [21]. Differently, a 43% increase in the seed productivity of *Brassica rapa* L. treated with sodium selenite was reported [22], while the increase in faba bean seed productivity was recorded only under a nano-Se supply, contrary to sodium selenate and selenite which inhibited seed production. Significant varietal differences were also found in the plant response to Se treatments [23]. According to Lyons’ report [22], the enhancement of the respiration rate in leaves and flowers as well as of the mitochondrial activity as a result of the Se supply positively affects pollen development leading to increased fertile seeds.

Furthermore, the effects of garlic extract application to plants in improving the chlorophyll, soluble sugar and antioxidant profile [4,24], including protecting due to its antibacterial, antimicrobial and anti-fungal properties [25,26] and stimulating growth, entail high prospects of the utilization of the mentioned extract in seed production. Allopathic benefits derived from garlic stem dry powder supplied to soil were also reported on lettuce growth and development [7]. Garlic extract can also positively affect plant productivity, as described in *Vicia faba* [27].

Based on the mentioned aspects, it is worth assessing the efficacy of the single and combined utilization of different biofortification-biostimulation agents, such as selenium and garlic extracts, particularly on seed-addressed plants. In this respect, the choice of lettuce is especially due to its wide diffusion worldwide, fast growth and high nutritional value also depending on genotype, as in most crops [28,29].

The aim of the present investigation was the evaluation of a single and joint foliar application of sodium selenate and garlic bulb extract on the seed productivity and seed quality of four lettuce cultivars, as well as on the yield and quality of mature plants grown from these seeds.

## 2. Results and Discussion

Compared to the soil Se supply, the foliar application of Se and garlic extract minimizes the soil’s microbiological and chemical effect on this treatment’s efficiency and induces a higher absorption rate, as reported about Se biofortification in various plant species [10,30].

### 2.1. Seed Quality

The results of the present investigation indicated that both the single and combined treatment of seed-addressed plants with the sodium selenate solution and garlic extract did not cause any significant effect on seed weight or germination energy and capacity (Table 1). The mean 1000-seed weight of the cultivars examined ranged between 1.04 and 1.30 g, with significantly higher values recorded for cvs. Bouquet and Cavalier, compared to cvs. Picnic and Moskovsky parnikovy. The latter two cultivars also demonstrated higher levels of germination energy than cv. Cavalier, whereas the germination capacity was not significantly affected by the cultivar.

A significant beneficial effect of Se and garlic extract treatments was recorded on the seed productivity of all the lettuce cultivars tested, compared to the control (Table 2).

**Figure 1:**
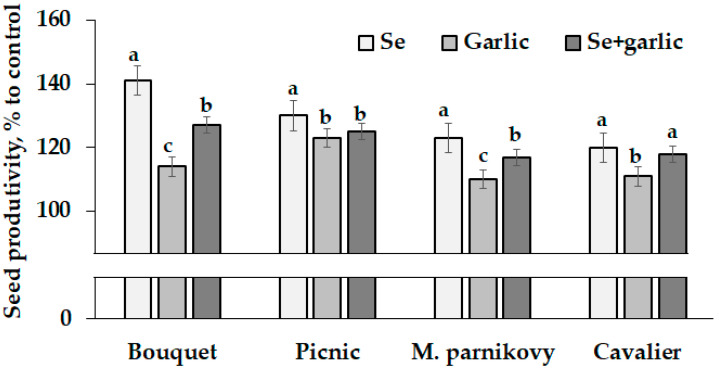
Changes in seed productivity of lettuce under Se/garlic application. Within each cultivar, values with the same letters do not differ statistically according to Duncan’s test at *p* < 0.05.

It is noteworthy that the greatest differences in the treatment response were recorded for cv. Bouquet, whose seed yield ranged from 2.2 to 3.1 g per plant and decreased according to the following sequence: selenate > (selenate + garlic) > garlic > control. The mentioned cultivar showed the highest increase (141%) in seed productivity, compared to the untreated control, under the selenate supply (Figure 1), though the other three cultivars tested displayed the same trend but with smaller differences versus the control plants. The obtained results indicate the possibility of a 110% to 141% seed productivity increase due to Se/garlic extract application. Figure 1 shows that the smallest changes in seed productivity due to the treatments applied were recorded for cv. Picnic (123–130%), with an increasing range in Cavalier (111–120%), Moskovsky parnikovy (110–123%) and Bouquet (114–141%). Notably, the significant varietal differences recorded in *Vicia faba* seed productivity under garlic extract application as well as the latter beneficial effect on plant performances may be connected with various garlic biologically active compounds, predominantly sulfur derivatives along with starch, vitamins and polyphenols [31].

Though sodium selenate and garlic extract did not have a synergistic effect on seed productivity, their joint application is beneficial for lettuce seed production and should be considered for the antioxidant and anti-viral protection of plants.

The comparison of the present results with the effect of Se on seed productivity in *Faba bean* [23], *Brassica rapa* [22] and rice [21] indicates the species importance in the plant response to different Se chemical forms and concentrations.

### 2.2. Seed Antioxidant Status

#### 2.2.1. Proline Accumulation

**Figure 2:**
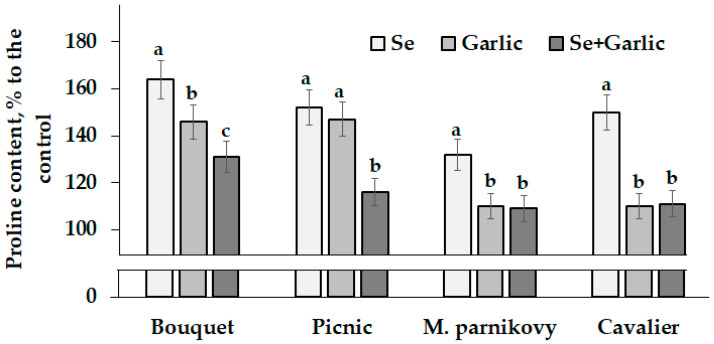
Changes in proline accumulation in lettuce seeds under Se/garlic application. Within each cultivar, values with the same letters do not differ statistically according to Duncan’s test at *p* < 0.05.

Seed viability and the efficiency of their storage greatly depend on the antioxidant status parameters. Among the latter, proline is involved in the plant antioxidant system and highly valued for its ability to enhance plant immunity [32]. The mentioned amino acid is known to be easily accumulated in seeds, providing energy for plant reproduction and increasing the level of antioxidant protection [33]. According to the literature reports, the foliar application of garlic extract to *Vicia faba* plants increases plant indole acetic acid content as well as seed yield and proline accumulation [24]. The results of the present investigation indicate the highest proline concentration increase in lettuce seeds under a sodium selenate supply (Table 3; Figure 2), which is consistent with the known phenomenon of Se’s beneficial effect on N metabolism and amino acid accumulation [10].

The varietal differences showed a significant increasing trend in the seed proline content from Picnic to Bouquet and Moskovsky parnikovy, reaching the highest value in cv. Cavalier, both in the control plants and those treated with sodium selenate and garlic extract (Table 3). Compared to the control, cvs. Bouquet and Picnic always showed the highest increase in proline content, with the highest gap recorded under the supply of garlic extract (Figure 2); the latter, singly or combined with Se, led to the lowest changes in the seed proline content in cvs. M. parnikovy and Cavalier (Figure 2). Generally, the joint Se/garlic extract application was less effective in increasing the proline levels in lettuce seeds, compared to their single treatments (Figure 2).

#### 2.2.2. Total Antioxidant Activity and Polyphenol Content

AOA increased in lettuce plants only under the Se supply in all cultivars, whereas the polyphenol content was not affected by any treatments, but only in cv. Moskovsky parnikovy garlic and the combination Se/garlic extract caused a reduction, compared to the control (Table 3).

#### 2.2.3. Se Accumulation

**Figure 3:**
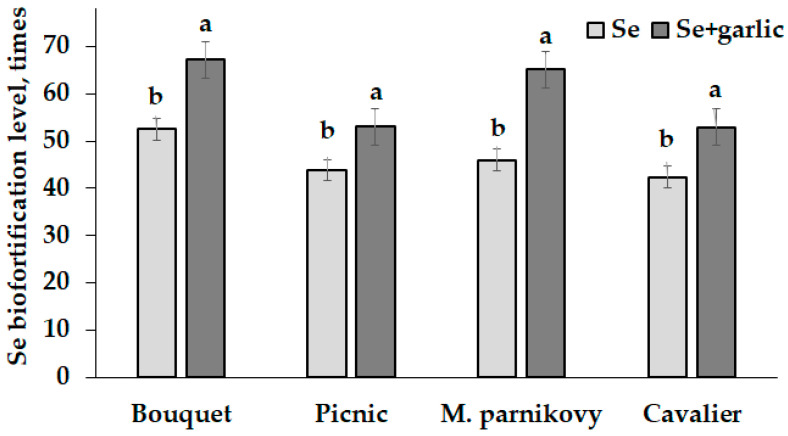
Selenium biofortification levels of lettuce seeds. Within each cultivar, values with the same letters do not differ statistically according to Duncan’s test at *p* < 0.05.

The biofortification of lettuce seed-addressed plants with Se resulted in a high accumulation of this element in seeds reaching concentrations of 4000 to 6000 µg Se kg^−1^ d.w. (Table 3). Interestingly, the garlic extract supply significantly stimulated the accumulation of Se in lettuce seeds (Figure 3). A similar phenomenon was previously recorded in *Vicia faba* fortified with sodium selenite under garlic peel extract treatment [20], but it is influenced by the following: the Se form (selenite instead of selenate); garlic tissue (garlic peel or bulb); garlic extraction method (ethanolic extract in [20] or water extract in the present work); and the objects of the Se/garlic treatment (seed soaking compared to foliar Se/garlic extract supply). In this respect, Nossie et al. [20] reported that the beneficial effect of garlic application is connected with high concentrations of antioxidants, mainly polyphenols and quercetin easily extracted by ethanol. Contrarily, water extraction used in the present work provided the predominant release of sulfur derivatives such as ajoenes, thiosulfinates, different dithiins and others [34]. The effect of selenium–sulfur competition on the efficiency of plant assimilation was recorded only in conditions of soil Se supply [35,36]. Contrarily, the foliar selenium supply elicited a synergism between the two mentioned elements, thus enhancing the plant yield and amino acids’ accumulation [37]. The results of the present research indicate that the garlic extract supply stimulated Se accumulation in lettuce seeds (Figure 3).

The literature reports highlight the importance of the Se/S balance in plants to promote growth and development [38]. Further investigations are necessary to clarify the Se-S relationships via the utilization of individual organic sulfur compounds isolated from garlic.

### 2.3. Health-Promoting Properties of Lettuce Seeds

Taking into account the high AOA activity and polyphenol concentrations in lettuce seeds, the remarkable nutritional value of lettuce seeds even rises under supplementation with Se/garlic extract. Indeed, seeds with a high Se content in plants singly or jointly treated with garlic extract have important health-promoting properties as a source of natural antioxidants, such as selenium, polyphenols, flavonoids and proline. In general, lettuce seeds are known for their sedative, antioxidant, cardio-protective, anti-inflammatory, anti-cancer and spermatozoa regulation properties. Seed proline and Se participate in protein biosynthesis, antioxidant defense and the maintenance of human immunity [39,40,41]. Seed polyphenols and flavonoids are highly valued due to the ability to protect the organism against cancer, cardiovascular disease and age-related degenerative diseases [35]. Selenium enhances immunity, optimizes fertility and protects the organism against viral, cardiovascular diseases and cancer [42]. Ten grams of lettuce seeds obtained from plants treated with Se/garlic extract can provide about 57–71% of the adequate daily Se consumption level equal to 70 µg/day [38].

### 2.4. Biometrical and Biochemical Characteristics of Mature Plants

**Figure 4:**
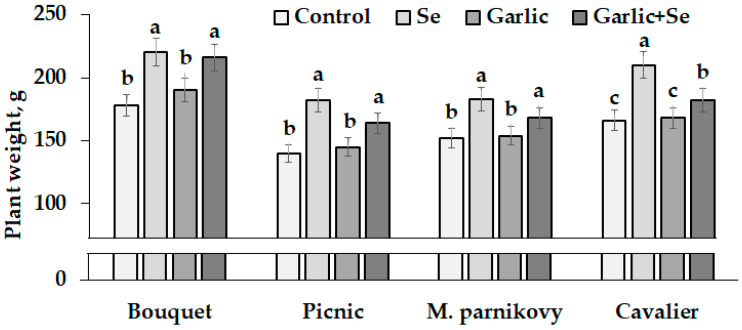
Lettuce weight of control and Se/garlic extract-treated plants. Within each cultivar, values with the same letters do not differ statistically according to Duncan’s test at *p* < 0.05.

The treatment of seed-addressed plants with Se/garlic extract affected both the quality of seeds and of mature plants. High levels of seed germination both without and with the Se and garlic extract supply indicate modified seed suitability for lettuce growth. Figure 4 demonstrates the significant beneficial effect that the use of biofortified seeds had on the plant biomass of all lettuce cultivars grown in the greenhouse. Despite varietal differences, the highest biomass was recorded for all plants grown from Se-modified seeds (after a single or joint application of Se and garlic extract). Contrarily, single garlic extract utilization in seed production did not significantly affect lettuce leaf weight. Among the cultivars examined, Bouquet showed the highest plant weight under the Se and garlic extract supply (Figure 4).

**Figure 5:**
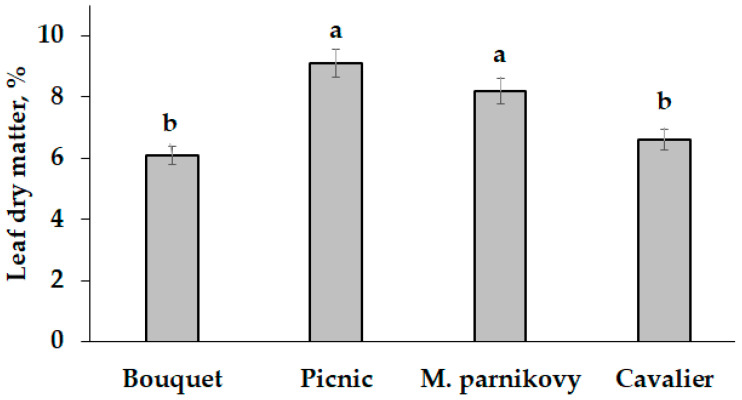
Varietal differences in the mean dry matter content of lettuce leaves. Values with the same letters do not differ statistically according to Duncan’s test at *p* < 0.05.

Contrarily, neither the single nor combined application of Se and garlic extract to seed-addressed plants affected the dry matter content in leaves of lettuce, which ranged from 6.1% (cv. Bouquet) to 9.1% (cv. Picnic) (Figure 5).

The photosynthetic pigment content positively correlated to the leaf biomass (r = 0.921; *p* < 0.001), which proves the importance of the beneficial Se effect on chlorophyll accumulation in plants [43].

**Figure 6:**
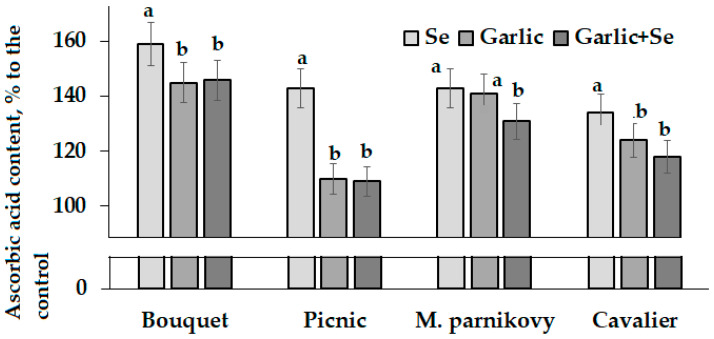
Differences in the ascorbic acid content in lettuce leaves grown from the seeds of modified seed-addressed plants. Within each cultivar, values with the same letters do not differ statistically according to Duncan’s test at *p* < 0.05.

Compared to the untreated control, leaf AOA generally increased upon Se/garlic extract treatments, whereas the total phenolic content was more affected by all the mentioned treatments in cv. Bouquet but showed controversial trends in the other three cultivars. Interestingly, the lettuce leaf AOA and TP values were close to the corresponding parameters in seeds (Table 3, Table 4 and Table 5), which is in accordance with the literature reports regarding the high antioxidant status of both lettuce leaves and seeds [39]. Contrary to AOA and TP, a significant increase in the ascorbic acid content due to Se application was recorded in all cultivars tested (Figure 6).

The maintenance of the high ascorbic acid content in agricultural crops is highly important both for consumer health and for enhancing plant growth and development. Indeed, ascorbic acid is essential for plants, animals and humans, is an important component of the plant antioxidant system, participates in cell division and growth, signal transduction and hormone biosynthesis and is a cofactor of several enzymes involved in photosynthesis [44].

**Figure 7:**
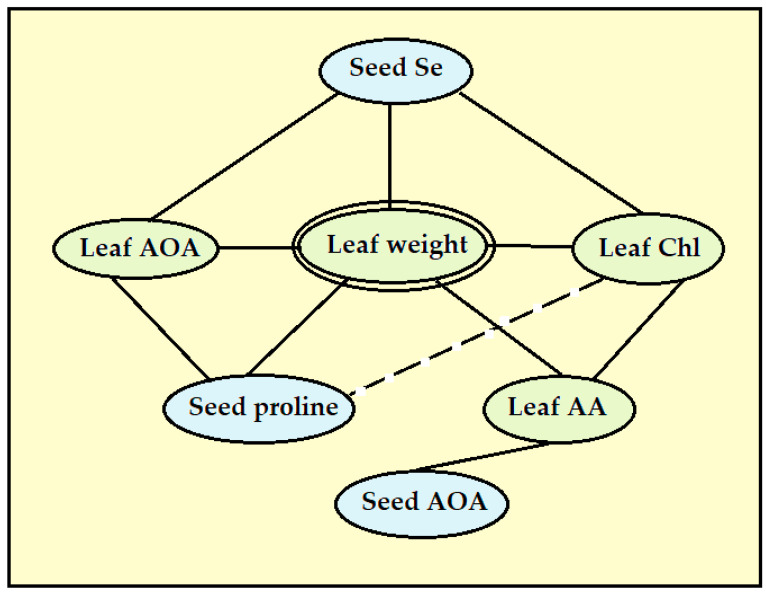
The most significant relationships between the leaf weight and antioxidant status of seeds and leaves.

The present results indicate a close relationship between the levels of the ascorbic acid in lettuce leaves and the leaf weight and chlorophyll content (Table 6, Figure 7). Furthermore, its concentration in lettuce leaves positively correlates with seed antioxidant activity (r = 0.635, *p* < 0.005; Table 6).

Seed quality mostly determines the yield and quality of mature plants. The analysis of correlations between the parameters tested demonstrated significant correlations between seed and leaf antioxidant status: (1) a beneficial effect of seed Se on leaf chlorophyll, the total antioxidant activity and leaf weight, (2) a similar beneficial effect of the seed proline content and (3) positive correlations between the leaf weight and leaf AOA, chlorophyll levels and ascorbic acid accumulation (Table 6, Figure 7). The lack of a significant correlation between the leaf proline levels, plant weight and nutritional value of mature plants, contrary to seed proline, suggests the significance of the latter parameter as a predictor of the mature plant yield and quality.

The revealed relationships between lettuce yield and quality with the corresponding characteristics of seeds suggest the importance of the seed plant treatment both for the seed productivity/seed quality and yield/quality of lettuce grown from these seeds.

## 3. Materials and Methods

### 3.1. Growing Conditions and Experimental Protocol

The research was conducted in 2022–2023 on lettuce (*Lactuca sativa* L.) at the experimental fields of the Federal Scientific Vegetable Center in Moscow (55°39.510 N, 37°12.230 E).

The experimental protocol was based on the factorial combination between 4 lettuce cultivars (Bouquet, Picnic, Moskovsky parnikovy, Cavalier) selected at the Federal Scientific Vegetable Center and 3 biofortification treatments (selenium, garlic extract, selenium + garlic extract) plus an untreated control. A split plot design with three replications was used for the treatment distribution in the field.

### 3.2. Seed Production

Plants addressed to seed production were grown in a greenhouse at 15 °C mean temperature and 70–80% relative humidity, starting from seed sowing in polystyrene trays on 8 April 2021 and 2022. The seedlings were planted in open field soil on 6 May in 1.4 m wide beds, with a spacing of 30 cm between the plants along the rows which were 45 cm apart. A loam sod podzolic soil was used for this experiment, with the following characteristics: pH 6.2, 2.12% organic matter, 1.32 mg-eq 100 g^−1^ hydrolytic acidity, 18.5 mg kg^−1^ mineral nitrogen, 21.3 mg kg^−1^ ammonium nitrogen, a sum of absorbed bases as much as 93.6%, 402 mg kg^−1^ mobile phosphorous, 198 mg kg^−1^ exchangeable potassium, 1 mg kg^−1^ S, 10.95 mg kg^−1^ Ca, 2.05 mg kg^−1^ Zn, 0.86 mg kg^−1^ B, 220 µg kg^−1^ d.w. Se, 7.65 mg kg^−1^ Ni, 0.22 mg kg^−1^ Cd, 1.6 mg kg^−1^ As, and 12.85 mg kg^−1^ Pb.

Prior to sowing, the soil was accurately ploughed at 40 cm depth, and during the growing season, hoeing and manual weeding were carried out according to the needs determined through constant monitoring. Fertilization was performed before transplanting, supplying 45 kg ha^−1^ N, 30 kg ha^−1^ P_2_O_5_ and 45 kg ha^−1^ K_2_O. Irrigation was activated when the soil humidity dropped to 80% of the available water capacity at 20 cm depth. The foliar treatment of plants with the sodium selenate solution (25 mg L^−1^), garlic extract and their combined application was performed twice on mature plants at the stage of marketable ripening before the inflorescence formation, on 27 May and 7 days later. In each treatment, 200 mL m^−2^ solution was necessary for intensive leaf watering. All treatments given to plants were carried out in the evening in dry weather conditions.

Garlic extract was prepared according to the Wafaa et al. method [45] via the homogenization and extraction of fresh garlic bulbs with distilled water (500 g:500 mL), the filtration of a mixture and a dilution of the resulting extract twice with water. Seed harvesting was performed manually on 10–12 August, via plant cutting 20 cm above the ground with subsequent ripening on a stem dryer. Seed threshing and cleaning were carried out manually on 25–27 August.

The mean values of the monthly temperature and rainfall during the crop cycles are presented in Table 7.

### 3.3. Lettuce Growth

Lettuce was planted on 4 May 2022 and 2023 in a greenhouse using drip irrigation, in a loam sod podzolic soil with the addition of lowland peat, pH 6.4, with a spacing of 20 cm between the plants along the rows which were 25 cm apart. Air temperature and relative humidity were maintained at 22/18 °C (day/night) and 65%, respectively. The experimental unit had a 5 m^2^ surface area, with 4 replicates.

The plants were harvested on 8–10 June at the stage of marketable ripening before inflorescence formation. After harvesting, leaves were rinsed with distilled water and dried with filter paper. Ten lettuce plants were sampled randomly from each cultivar to measure the morphologic traits. Part of the fresh leaves was used for the determination of the ascorbic acid and chlorophyll content, and the remaining leaf was dried and used for the analysis of the total antioxidant activity and the concentration of polyphenols and proline.

### 3.4. Sample Preparation

#### 3.4.1. Dry Matter

The dry matter was assessed gravimetrically by drying the samples in an oven at 70 °C until constant weight.

#### 3.4.2. Selenium

The selenium content in seeds was analyzed using the fluorimetric method previously described for tissues and biological fluids [46]. Dried homogenized samples were digested via heating with a mixture of nitric and perchloric acids, a subsequent treatment of samples with a solution of 6 N HCl to reduce selenate (Se^6+^) to selenite (Se^4+^) and the formation of a complex between Se^4+^ and 2,3-diaminonaphtalene. Selenium concentration was calculated by recording the piazoselenol fluorescence value in hexane at 519 nm λ emission and 376 nm λ excitation. Each determination was performed in triplicate. The precision of the results was verified using the Mitsuba reference standard of Se-fortified stem powder in each determination, with a Se concentration of 1865 µg kg^−1^ (Federal Scientific Vegetable Center).

#### 3.4.3. Total Polyphenols (TPs)

The total polyphenols were determined in 70% ethanol extracts of samples using the Folin–Ciocâlteu colorimetric method as previously described [47]. Half a gram of seed/leaf dry homogenates was extracted with 20 mL of 70% ethanol/water at 80 °C for 1 h. The mixture was cooled down and quantitatively transferred to a volumetric flask, and the volume was adjusted to 25 mL. The latter mixture was filtered through filter paper, and 1 mL of the resulting solution was transferred to a 25 mL volumetric flask, to which 2.5 mL of saturated Na_2_CO_3_ solution and 0.25 mL of diluted (1:1) Folin–Ciocâlteu reagent were added. The volume was brought to 25 mL with distilled water. One hour later, the solutions were analyzed through a spectrophotometer (Unico 2804 UV, Suite E Dayton, Newark, NJ, USA), and the concentration of polyphenols was calculated according to the absorption of the reaction mixture at 730 nm. As an external standard, 0.02% gallic acid was used. The results were expressed as mg of gallic acid equivalent per g of dry weight (mg GAE g^−1^ d.w).

#### 3.4.4. Antioxidant Activity (AOA)

The antioxidant activity of lettuce leaves and seeds was assessed on 70% ethanolic extracts of dry samples using a redox titration method [47]. The values were expressed in mg gallic acid equivalents (mg GAE g^−1^ d.w.).

#### 3.4.5. Ascorbic Acid

The ascorbic acid content was determined by the visual titration of leaf extracts in 3% trichloracetic acid with a sodium 2.6-dichlorophenol indophenolate solution (Tillman’s reagent) using a method of visual titration [48].

#### 3.4.6. Proline

Proline determination was conducted using a 3% sulfur salicylic extract of dry homogenized seed/leaf via the reaction with a ninhydrin reagent in acetic acid, as described by Ouertani et al. [49]. After heating at 95 °C for 1 h, the probes were cooled and extracted with toluene. Proline content was calculated using the absorption value of the extract at 505 nm and a calibration curve with 5 different proline (Merck) concentrations.

#### 3.4.7. Photosynthetic Pigments

Chlorophyll a, chlorophyll b and carotene content were assessed spectrophotometrically by a spectrophotometer Unico 2804 UV, using a 96% ethanol extract of fresh leaf samples and the equations developed by Lichtenthaler [50]:

Ch-a = 13.36A_664_ − 5.19A_649_;

Ch-b = 27.43A_649_ − 8.12A_664_;

C c = (1000A_470_ − 2.13 Ch-a − 87.63 Ch-b)/209;

where A = absorbance, Ch-a = chlorophyll a, Ch-b = chlorophyll b and C c = carotene.

### 3.5. Statistical Analysis

Data were processed by analysis of variance, and mean separations were performed through Duncan’s multiple range test, with reference to the 0.05 probability level, using the SPSS software version 28.

## 4. Conclusions

The present investigation provides innovative results regarding the efficiency of selenium/garlic extract application to enhance the lettuce seed productivity and quality of both the seeds and mature plants grown from these seeds. The lettuce seeds obtained from seed-addressed plant biofortification with Se, supplied singly or in combination with garlic extract, can be considered as a new functional food with high levels of antioxidants, nutrients and Se. The mentioned lettuce seeds provide an enhanced plant yield, antioxidant status and Se content. The beneficial effect of garlic extract on seed Se accumulation is especially important to increase Se biofortification efficiency. Nevertheless, further investigations are needed to unveil the mechanisms related to Se–garlic extract synergism.

## Figures and Tables

**Table 1:** Mean values of seed weight and germination energy and capacity of control and Se/garlic extract-treated plants.

Parameter	Cultivar	M ± SD	CV (%)	Range
Weight of 1000 seeds (g)	Bouquet	1.29 ± 0.01 a	0.80	1.28–1.30
	Picnic	1.12 ± 0.06 b	5.36	1.04–1.14
	M. parnikovy	1.19 ± 0.02 b	1.68	1.16–1.20
	Cavalier	1.29 ± 0.01 a	0.80	1.28–1.30
Germination energy (%)	Bouquet	91.5 ± 1.3 ab	1.42	90–93
	Picnic	94.0 ± 2.2 a	2.34	91–95
	M. parnikovy	94.0 ± 1.7 a	1.81	91–97
	Cavalier	90.3 ± 1.7 b	1.88	88–92
Germination capacity (%)	Bouquet	95.8 ± 1.7 a	1.77	94–98
	Picnic	94.8 ± 2.2 a	2.32	92–97
	M. parnikovy	92.9 ± 3.7 а	3.98	86–96
	Cavalier	93.5 ± 1.3 a	1.39	91–95

Within each parameter and column, values with the same letters do not differ statistically according to Duncan’s test at *p* < 0.05.

**Table 2:** Seed productivity of lettuce cultivars.

Cultivar	Control	Se	Garlic	Garlic + Se
Bouquet	2.2 ± 0.2 c	3.1 ± 0.2 a	2.5 ± 0.2 bc	2.8 ± 0.2 ab
Picnic	4.0 ± 0.3 b	5.0 ± 0.4 a	4.9 ± 0.4 a	5.2 ± 0.4 a
M. parnikovy	3.0 ± 0.2 b	3.7 ± 0.3 a	3.3 ± 0.2 ab	3.5 ± 0.2 a
Cavalier	2.8 ± 0.2 b	3.3 ± 0.2 a	3.0 ± 0.2 a	3.1 ± 0.2 ab

The values are expressed in g per plant. Along each line, values with the same letters do not differ significantly according to Duncan’s test at *p* < 0.05.

**Table 3:** Antioxidant status of lettuce seeds.

Parameter	Cultivar	Control	Selenium	Garlic	Garlic + Se
Proline (mg g^−1^ d.w.)	Bouquet	1.08 ± 0.05 d	1.77 ± 0.07 a	1.58 ± 0.07 b	1.41 ± 0.05 c
	Picnic	0.81 ± 0.04 b	1.23 ± 0.06 a	1.19 ± 0.04 a	0.94 ± 0.04 b
	M. parnikovy	1.25 ± 0.06 c	1.65 ± 0.07 a	1.37 ± 0.05 b	1.36 ± 0.05 bc
	Cavalier	1.43 ± 0.07 c	2.15 ± 0.08 a	1.58 ± 0.07 b	1.59 ± 0.07 b
	M ± SD	1.14 ± 0.29	1.70 ± 0.38	1.43 ± 0.21	1.33 ± 0.28
	CV (%)	25.4	22.4	14.7	21.1
AOA (mg-GAE g^−1^ d.w.)	Bouquet	22.9 ± 0.9 b	25.7 ± 0.9 a	22.0 ± 0.8 b	21.3 ± 0.8 b
	Picnic	22.3 ± 0.8 b	26.3 ± 0.9 a	23.0 ± 0.8 b	22.5 ± 0.8 b
	M. parnikovy	20.2 ± 0.7 b	24.2 ± 0.8 a	18.5 ± 0.6 c	17.5 ± 0.5 c
	Cavalier	19.6 ± 0.6 b	22.1 ± 0.7 a	18.6 ± 0.6 b	18.5 ± 0.6 b
	M ± SD	21.2 ± 1.6	24.6 ± 1.9	20.5 ± 2.3	20.0 ± 2.3
	CV (%)	7.5	7.7	11.2	11.5
TP (mg-GAE g^−1^ d.w.)	Bouquet	21.5 ± 1.0 a	22.4 ± 1.0 a	21.1 ± 1.0 a	20.5 ± 0.9 a
	Picnic	21.6 ± 1.0 a	23.5 ± 1.1 a	23.0 ± 1.1 a	21.9 ± 1.0 a
	M. parnikovy	19.2 ± 0.9 ab	21.0 ± 0.9 a	19.0 ± 0.9 b	16.0 ± 0.7 c
	Cavalier	12.2 ± 0.5 a	12.5 ± 0.5 a	13.3 ± 0.6 a	12.2 ± 0.5 a
	M ± SD	18.6 ± 4.4	19.9 ± 5.0	19.1 ± 4.2	17.7 ± 4.4
	CV (%)	23.7	25.1	22.0	24.9
Selenium (µg kg^−1^ d.w.)	Bouquet	106 ± 9 c	5138 ± 420 b	93 ± 8 c	6245 ± 511 a
	Picnic	116 ± 11 c	4357 ± 395 b	115 ± 10.0 c	5569 ± 455 a
	M. parnikovy	89 ± 8 c	4092 ± 324 b	79 ± 7 c	5146 ± 487 a
	Cavalier	110 ± 10 c	4240 ± 388 b	104 ± 9 c	5469 ± 442 a
	M ± SD	115 ± 28	4457 ± 467	98 ± 15	5607 ± 462
	CV (%)	24.3	10.5	15.3	8.2

AOA: total antioxidant activity; TP: total polyphenol content. Along each line, values with the same letters do not differ significantly according to Duncan’s test at *p* < 0.05.

**Table 4:** Photosynthetic pigments in lettuce plants as affected by Se/garlic extract treatments.

Parameter	Cultivar	Control	Selenate	Garlic	Garlic + Se
Chl a (mg g^−1^ f.w.)	Bouquet	0.71 ± 0.02 b	0.78 ± 0.03 a	0.66 ± 0.02 c	0.70 ± 0.02 b
	Picnic	0.65 ± 0.02 c	0.78 ± 0.03 a	0.59 ± 0.01 d	0.70 ± 0.02 b
	M. parnikovy	0.67 ± 0.02 b	0.81 ± 0.04 a	0.69 ± 0.02 b	0.79 ± 0.03 a
	Cavalier	0.64 ± 0.02 c	0.86 ± 0.04 a	0.59 ± 0.01 d	0.70 ± 0.02 b
	M ± SD	0.66 ± 0.03 b	0.81 ± 0.04 a	0.63 ± 0.05 b	0.72 ± 0.04 b
Chl b (mg g^−1^ f.w.)	Bouquet	0.38 ± 0.01 b	0.44 ± 0.01 a	0.36 ± 0.01 b	0.42 ± 0.01 a
	Picnic	0.37 ± 0.01 b	0.41 ± 0.01 a	0.33 ± 0.01 c	0.41 ± 0.01 a
	M. parnikovy	0.38 ± 0.01 c	0.47 ± 0.01 a	0.42 ± 0.01 b	0.43 ± 0.01 b
	Cavalier	0.39 ± 0.01 b	0.48 ± 0.01 a	0.36 ± 0.01 c	0.39 ± 0.01 b
	M ± SD	0.38 ± 0.01 b	0.45 ± 0.03 a	0.37 ± 0.03 b	0.41 ± 0.02 ab
Total chl (mg g^−1^ f.w.)	Bouquet	1.09 ± 0.04 bc	1.22 ± 0.05 a	1.02 ± 0.04 c	1.12 ± 0.04 b
	Picnic	1.02 ± 0.04 b	1.19 ± 0.04 a	0.92 ± 0.04 b	1.11 ± 0.04 a
	M. parnikovy	1.05 ± 0.04 b	1.28 ± 0.05 a	1.11 ± 0.04 b	1.22 ± 0.04 a
	Cavalier	1.03 ± 0.04 bc	1.34 ± 0.05 a	0.95 ± 0.04 c	1.09 ± 0.04 b
	M ± SD	1.04 ± 0.03 c	1.26 ± 0.06 a	1.00 ± 0.08 c	1.14 ± 0.05 b
Carotene (mg g^−1^ f.w.)	Bouquet	0.13 ± 0.01 b	0.16 ± 0.01 a	0.14 ± 0.01 ab	0.16 ± 0.01 a
	Picnic	0.14 ± 0.01 bc	0.18 ± 0.01 a	0.13 ± 0.01 c	0.15 ± 0.01 b
	M. parnikovy	0.15 ± 0.01 c	0.18 ± 0.01 a	0.16 ± 0.01 bc	0.17 ± 0.01 ab
	Cavalier	0.14 ± 0.01 b	0.16 ± 0.01 a	0.13 ± 0.01 c	0.17 ± 0.01 a
	M ± SD	0.14 ± 0.01 b	0.17 ± 0.01 a	0.14 ± 0.01 b	0.16 ± 0.01 ab

Along each line, values with the same letters do not differ significantly according to Duncan’s test at *p* < 0.05.

**Table 5:** Antioxidant status in lettuce mature plants as affected by Se/garlic extract treatments.

Parameter	Cultivar	Control	Selenium	Garlic	Garlic + Se
Proline (mg g^−1^ d.w.)	Bouquet	2.02 ± 0.08 a	2.01 ± 0.08 a	1.88 ± 0.07 a	1.84 ± 0.07 b
	Picnic	2.17 ± 0.09 a	1.82 ± 0.07 b	1.75 ±0.07 b	1.75 ± 0.07 b
	M. parnikovy	2.13 ± 0.08 a	2.00 ± 0.08 ab	2.02 ± 0.08 ab	1.86 ± 0.07 b
	Cavalier	1.99 ± 0.08 a	1.79 ± 0.07 a	1.69 ± 0.07 a	1.66 ± 0.07 a
	M ± SD	2.08 ± 0.08 a	1.91 ± 0.11 ab	1.84 ± 0.14 b	1.78 ± 0.09 b
AOA (mg-GAE g^−1^ d.w.)	Bouquet	25.2 ± 0.8 b	28.7 ± 0.9 a	25.7 ± 0.8 b	27.7 ± 0.9 a
	Picnic	21.6 ± 0.7 c	26.3 ± 0.9 ab	24.6 ± 0.8 b	28.2 ± 0.9 a
	M. parnikovy	23.1 ± 0.7 b	28.3 ± 0.9 a	27.1 ± 0.9 a	28.7 ± 0.9 a
	Cavalier	24.1 ± 0.7 b	27.8 ± 0.9 a	27.8 ± 0.9 a	28.3 ± 0.9 a
	M ± SD	23.5 ± 1.5 b	27.8 ± 1.0 a	26.3 ± 1.4 ab	28.2 ± 0.4 a
TP (mg-GAE g^−1^ d.w.)	Bouquet	17.3 ± 0.7 b	19.4 ± 0.9 a	20.2 ± 0.9 a	20.6 ± 0.9 a
	Picnic	17.5 ± 0.7 b	18.4 ± 0.8 ab	19.9 ± 0.9 a	17.9 ± 0.8 b
	M. parnikovy	18.4 ± 0.8 a	19.9 ± 0.9 a	19.0 ± 0.8 a	19.0 ± 0.8 a
	Cavalier	17.1 ± 0.7 b	20.3 ± 0.9 a	19.8 ± 0.9 a	18.4 ± 0.8 b
	M ± SD	17.6 ± 0.6 b	19.5 ± 0.8 a	19.7 ± 0.5 a	18.9 ± 1.1 ab
AA (mg 100 g^−1^ f.w.)	Bouquet	11.5 ± 0.9 b	18.3 ± 1.2 a	16.7 ± 1.1 a	16.8 ± 1.1 a
	Picnic	17.8 ± 1.2 b	25.4 ± 1.3 a	19.5 ± 1.3 b	19.4 ± 1.3 b
	M. parnikovy	12.3 ± 0.9 b	17.6 ± 1.2 a	17.4 ± 1.2 a	16.1 ± 1.1 a
	Cavalier	10.3 ± 0.7 c	15.8 ± 1.1 a	12.8 ± 1.0 b	12.2 ± 0.9 bc
	M ± SD	12.9 ± 3.3 b	19.3 ± 4.2 a	16.6 ± 2.8 b	16.1 ± 3.0 b

AOA: total antioxidant activity; TP: total polyphenol content; AA: ascorbic acid. Along each line, values with the same letters do not differ significantly according to Duncan’s test at *p* < 0.05.

**Table 6:** Correlations between leaf and seed parameters.

	Seed Proline	Seed Se	Seed AOA	Plant Weight	Leaf AA	Leaf AOA	Chl
Seed productivity	−0.401	0.250	0.379	−0.358	0.758 a	0.007	0.023
Seed proline		0.298	−0.030	0.868 a	0.482	0.815 a	0.811 a
		Seed Se	0.196	0.621 c	0.346	0.730 a	0.672 b
		Seed AOA		0.340	0.635 c	−0.051	0.292
			Plant weight		0.665 b	0.924 a	0.921 a
				Ascorbic acid		0.727 a	0.743 a
				Leaf AOA			0.945 a

AOA: total antioxidant activity, AA: ascorbic acid, Chl: total chlorophyll; significance of correlations: (a) *p* < 0.001; (b) *p* < 0.002; and (c) *p* < 0.005.

**Table 7:** Mean values of monthly temperature and rainfall in 2021 and 2022.

Month	Temperature (°C)		Rainfall (mm)	
	2021	2022	2021	2022
May	13.8	10.7	81	61
June	21.8	18.9	20	42
July	22.0	20.7	38	91
August	19.4	21.9	36	4

## Data Availability

Data are contained within the article.
